# Therapeutic overexpression of miR-92a-2-5p ameliorated cardiomyocyte oxidative stress injury in the development of diabetic cardiomyopathy

**DOI:** 10.1186/s11658-022-00379-9

**Published:** 2022-10-08

**Authors:** Manli Yu, Yangyong Sun, Xinghua Shan, Fan Yang, Guojun Chu, Qian Chen, Lin Han, Zhifu Guo, Guokun Wang

**Affiliations:** 1 Department of Cardiology, Changhai Hospital, Naval Medical University, Shanghai, 200433 China; 2 Department of Cardiovascular Surgery, Institute of Cardiac Surgery, Changhai Hospital, Naval Medical University, 168 Changhai Road, Shanghai, 200433 China

**Keywords:** Diabetic cardiomyopathy, Cardiomyocytes, Oxidative stress, MicroRNA, MAP kinase interacting serine/threonine kinase 2

## Abstract

**Background:**

Diabetic cardiomyopathy (DCM) results from pathological changes in cardiac structure and function caused by diabetes. Excessive oxidative stress is an important feature of DCM pathogenesis. MicroRNAs (miRNAs) are key regulators of oxidative stress in the cardiovascular system. In the present study, we screened for the expression of oxidative stress-responsive miRNAs in the development of DCM. Furthermore, we aimed to explore the mechanism and therapeutic potential of miR-92a-2-5p in preventing diabetes-induced myocardial damage.

**Methods:**

An experimental type 2 diabetic (T2DM) rat model was induced using a high-fat diet and low-dose streptozotocin (30 mg/kg). Oxidative stress injury in cardiomyocytes was induced by high glucose (33 mmol/L). Oxidative stress-responsive miRNAs were screened by quantitative real-time PCR. Intervention with miR-92a-2-5p was accomplished by tail vein injection of agomiR in vivo or adenovirus transfection in vitro.

**Results:**

The expression of miR-92a-2-5p in the heart tissues was significantly decreased in the T2DM group. Decreased miR-92a-2-5p expression was also detected in high glucose-stimulated cardiomyocytes. Overexpression of miR-92a-2-5p attenuated cardiomyocyte oxidative stress injury, as demonstrated by increased glutathione level, and reduced reactive oxygen species accumulation, malondialdehyde and apoptosis levels. MAPK interacting serine/threonine kinase 2 (MKNK2) was verified as a novel target of miR-92a-2-5p. Overexpression of miR-92a-2-5p in cardiomyocytes significantly inhibited MKNK2 expression, leading to decreased phosphorylation of p38-MAPK signaling, which, in turn, ameliorated cardiomyocyte oxidative stress injury. Additionally, diabetes-induced myocardial damage was significantly alleviated by the injection of miR-92a-2-5p agomiR, which manifested as a significant improvement in myocardial remodeling and function.

**Conclusions:**

miR-92a-2-5p plays an important role in cardiac oxidative stress, and may serve as a therapeutic target in DCM.

**Graphical Abstract:**

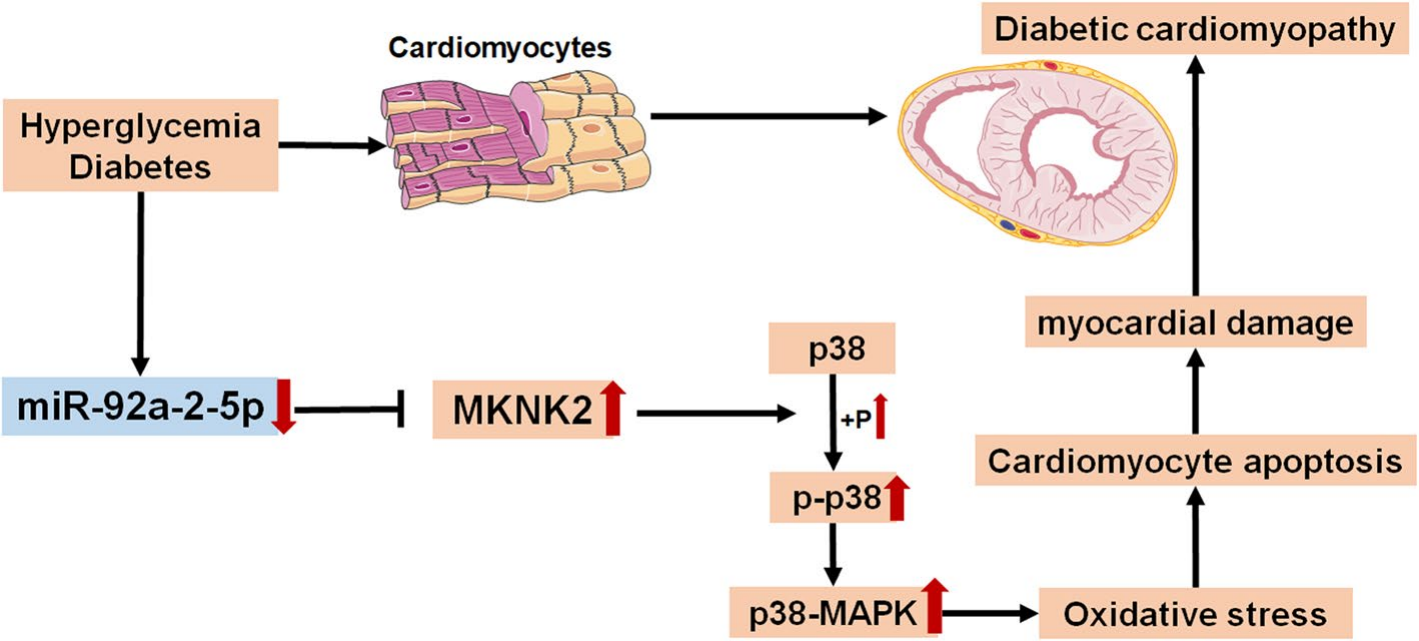

**Supplementary Information:**

The online version contains supplementary material available at 10.1186/s11658-022-00379-9.

## Background

Diabetic cardiomyopathy (DCM) manifests as heart failure symptoms without evidence of other cardiovascular diseases. It is characterized by diastolic dysfunction, described as impaired left ventricular relaxation, resulting in increased pressures and decreased end-diastolic filling [1, 2]. The development of cardiac dysfunction and heart failure in patients with DCM is independently associated with diabetes mellitus (DM), which is expected to affect more than 592 million people by 2035 [3, 4]. Half of the patients with diabetes diagnosed at preclinical or clinical stages are affected by DCM [5]. Moreover, patients with diabetes develop heart failure with a worse prognosis, more than twice that of patients without diabetes [6, 7]. The pathological mechanisms of DCM are complex and include metabolic disorders, oxidative stress, myocardial fibrosis, mitochondrial dysfunction, cardiomyocyte apoptosis, and chronic local inflammation [8, 9]. However, the underlying pathogenesis of DCM has not been thoroughly elucidated.

Oxidative stress is an important pathological and physiological process in diabetes pathogenesis. In patients with diabetes, the endogenous system of antioxidants, including superoxide dismutase, catalase and glutathione peroxidase, is adversely affected by hyperglycemia, resulting in progressive oxidative stress damage [10, 11]. The production of reactive oxygen species (ROS) increases in patients with diabetes and causes various diabetic complications. Over-accumulation of ROS activates mitochondrial apoptotic pathways, p38 mitogen-activated protein kinase (MAPK) pathway, and p53 apoptotic pathway, thereby promoting cardiomyocyte apoptosis and negatively influencing cardiac structure and function, ultimately leading to heart failure in DCM [12, 13]. Current antioxidative treatments for DCM are ineffective; hence, there is an urgent need to develop better treatments for DCM.

MicroRNAs (miRNAs) are an important class of endogenous small non-coding RNAs that regulate the expression of target genes at the post-transcriptional level. MiRNAs are fine-tuning regulators of diverse biological processes, including embryonic development, metabolism, inflammation, and cancer [14, 15]. In mammalian cells, miRNAs exert their suppressive function in a sequence-specific manner generally by binding to partially complementary sequences in the 3’-UTR of the targeted mRNAs [16]. An increasing number of studies have demonstrated that miRNAs play essential roles in the pathogenesis of cardiovascular diseases, such as myocardial infarction, valval calcification, and arrhythmias [17–19]. Some miRNAs have been identified as regulators of oxidative stress in the cardiovascular system by targeting ROS generators, antioxidant signaling pathways, and selected antioxidant effectors [20, 21]. Recently, miRNA-based therapies for patients with cardiovascular diseases have been preclinically developed [22], making functional exploration of miRNAs particularly important. In the present study, we identified the role of oxidative stress-responsive miRNAs in the development of DCM. Furthermore, we aimed to explore the mechanism and therapeutic potential of miR-92a-2-5p in preventing diabetes-induced in myocardial damage.

## Methods

### Experimental T2DM rat model

Adult male Sprague–Dawley (SD) rats (200 ± 20 g) were purchased from SLAC Laboratory Animal Co., Ltd. (Shanghai, China), and kept under regular conditions (22–55 °C, 50–60% humidity, and a 12 h light/dark cycle) for 1 week. The experimental T2DM rat model was induced by a high-fat diet (HFD) and low-dose streptozotocin (STZ), as previously reported [23]. Briefly, rats received HFD for 4 weeks and then received a single tail vein injection of STZ (30 mg/kg. Sigma, St. Louis, USA) dissolved in citrate buffer (pH 4.5). Blood glucose levels were measured weekly using a glucometer (Roche Diagnostics, Germany) after the STZ injection. Rats with a random blood glucose level over 16.7 mmol/L and impaired insulin sensitivity were considered T2DM rat models for further investigation. Rats in the control group were fed regular chow and injected with the same dose of citrate buffer.

### Cardiac echocardiography assessment

Two-dimensional guided M-mode echocardiography was conducted using an echocardiogram (VisualSonics, Canada) equipped with a 30-MHz high-frequency scan head. Upon successful anesthetization, an M mode cursor is positioned perpendicular to the interventricular septum and posterior wall of the left ventricle at the level of the papillary muscles via parasternal long-axis view. The M-mode image was obtained at a sweep speed of 100 mm/s.

### Histological analysis

The harvested hearts were immediately fixed in 4% paraformaldehyde, and embedded in paraffin. Several transverse sections were cut from midventricular sections. After deparaffinization and rehydration, the sections were stained with hematoxylin and eosin (H&E) for routine examination, or Masson trichrome for collagen fiber deposition examination.

### Immunohistochemical analysis

Immunohistochemical staining was performed as described previously [24]. Briefly, deparaffinized and rehydrated sections were subjected to antigen retrieval in a warm citric acid/sodium citrate buffer (pH 6.0). After blocking the endogenous peroxidases, the sections were incubated with a diluted primary antibody (1:100 dilution) overnight, followed by incubation with a secondary antibody. The staining signal was developed using a 3,3′-diaminobenzidine (DAB) development kit (Beyotime, China).

### Primary cardiomyocyte culture and treatment

Primary cardiomyocytes were isolated and cultured as described previously [25]. Briefly, ventricular tissues were harvested from 2-day-old neonatal Sprague–Dawley rats (SLAC, China) and minced into pieces. The tissues were then incubated overnight in a balanced salt solution containing collagenase (1 mg/mL Sigma, USA) at 4 °C. After passing through a cell strainer, cardiomyocytes were isolated using the differential preplating method, and cultured in M199 medium supplemented with 10% fetal bovine serum and bromodeoxyuridine (BrdU. 0.1 mmol/L) for 48 h. The identification and purity of cardiomyocytes were determined by an immunofluorescence assay with α-actinin antibody (Abcam, UK). Cardiomyocyte oxidative stress was induced by high glucose (33 mmol/L) treatment for 48 h. The same dose of mannitol was used as an osmotic control for high glucose levels.

### Intracellular ROS detection assay

Intracellular ROS levels were determined using 2′,7′-dichlorofluorescein diacetate (DCFH-DA. Beyotime). Briefly, cardiomyocytes were seeded in 24-well plates, and treated with high glucose (33.0 mmol/L). After washing, cardiomyocytes were incubated with DCFH-DA (10 μmol/L) for 20 min at 37 °C. Fluorescent signals were observed under an inverted fluorescence microscope (Olympus, Japan) or measured using a microplate reader (BioTek, USA) at 535 nm.

### Apoptosis assay

Cardiomyocyte apoptosis was determined using a TUNEL assay according to the manufacturer’s instruction. Briefly, isolated cardiomyocytes were seeded in 6-well plate and stimulated with high glucose for 48 h. After fixing in 4% formaldehyde for 30 min, the cells were incubated in phosphate-buffered saline containing 0.1% Triton X-100, then stained with TUNEL working solution at room temperature for 60 min, and incubated with 4’,6-diamidino-2-phenylindole (DAPI. 5 μg/mL) for 5 min. TUNEL-positive cells were counted in 5 random fields under a fluorescence microscope. The apoptosis level was evaluated according to the ratio of TUNEL-positive nuclei to DAPI-positive nuclei.

### Construction of recombinant adenovirus

Recombinant adenoviruses were constructed using the AdMax system (Hanbio Biotechnology, China) according to the manufacturer’s instructions. After purification by CsCl density-gradient centrifugation, the adenovirus titer in plaque-forming units (pfu) was determined by a plaque formation assay in 293 cells. The optimal multiplicity of infection of the adenovirus in cardiomyocytes was determined to be 20 pfu/cell.

### Quantitative real-time PCR (qRT-PCR) assay

Total RNA was isolated from tissues or cells using the MiniBEST Universal RNA Extraction Kit (TaKaRa, China) according to the manufacturer’s protocol. After quantifying using a spectrophotometer, equal amounts of RNA samples were reverse-transcribed using a PrimeScript RT Reagent Kit (TaKaRa) with a special stem-loop primer. qRT-PCR was performed on a LightCycler 480 II PCR system (Roche, Switzerland)) with TB Green Premix Ex TaqII (TaKaRa). The relative expression of miRNAs was analyzed using the 2^(−ΔΔCt)^ method. RNU6b was used as a loading control. Primer sequences are listed in Additional file 1: Table S1.

### Western blot analysis

The tissues or cells were lysed in RIPA lysis buffer plus cocktail (Thermo Fisher Scientific, USA). The concentration of extracted protein was quantified using a bicinchoninic acid (BCA) assay (Beyotime, China). Equal amounts of protein were separated by 10% sodium dodecyl sulfate–polyacrylamide gel electrophoresis (SDS-PAGE) and transferred to a polyvinylidene fluoride (PVDF) membrane (Pall, USA). The membranes were blocked with 5% (w/v) fat-free milk in Tris-buffered saline and Tween-20 at room temperature for 60 min. After washing, the membrane was incubated with primary antibody (1: 1000 dilution) at 4 °C overnight, followed by peroxidase-conjugated secondary antibody at room temperature for 1 h, and finally detected with ECL Plus Western Blotting Substrate (Thermo Fisher Scientific) on ChemiDoc MP system (Bio-Rad, USA). Quantification of immunoblot density was performed using ImageJ software. β-actin was used as a loading control. Information on the antibodies is presented in Additional file 1: Table S2.

### Bioinformatics analysis

Potential targets of miR-92a-2-5p were predicated using MicroRNA Target Prediction Database and TargetScan algorithm [26, 27]. The RNAhybrid algorithm was used to analyze the complementary matching and minimum free energy of hybridization between miR-92a-2-5p and the binding sites in the 3’-untranslated region (3’-UTR) of target mRNA [28].

### Dual‐luciferase reporter gene assay

The wild-type or mutant 3′-UTR of MKNK2 was synthesized and subcloned into luciferase reporter plasmid. The recombinant reporter plasmid, Renilla reference plasmid, and miR-92a-2-5p mimic were cotransfected into HEK293T cells. The cells were harvested 48 h after transfection. Luciferase activity was detected using a dual luciferase reporter assay kit (Beyotime).

### Statistical analysis

Statistical analyses were performed using SPSS v22.0 software. The differences between two groups were analyzed by Student’s t-test or Mann–Whitney U test. The differences between multiple groups were analyzed by one-way ANOVA followed by post hoc Tukey test or one-way ANOVA followed by Dunnett’s T3 test. Statistical significance was set at p < 0.05.

## Results

### Oxidative stress-responsive miRNAs were differentially expressed in the DCM rat heart

**Fig. 1:**
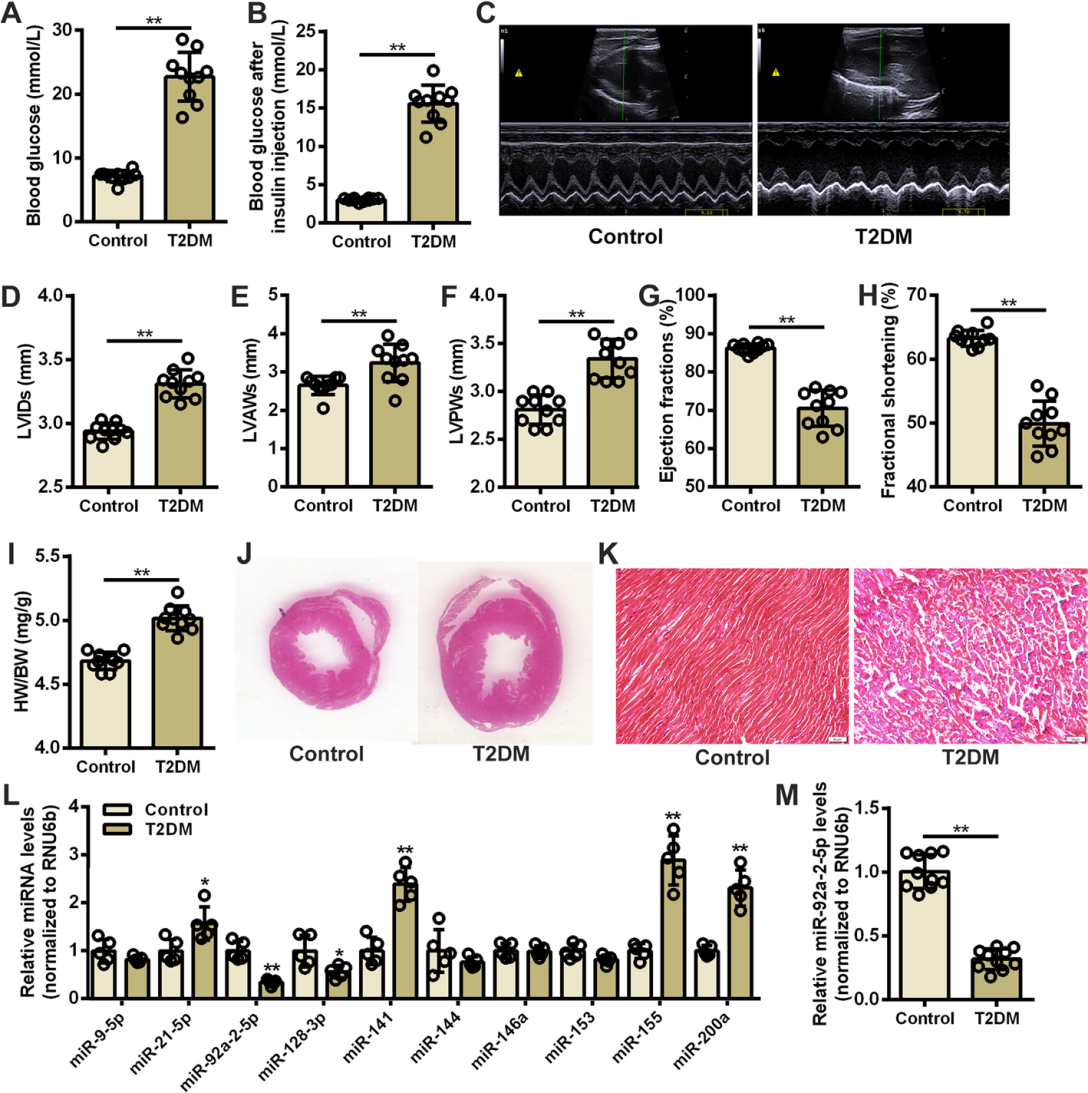
Oxidative stress-responsive miRNAs were differentially expressed in DCM rat hearts. **A** Detection of random blood glucose levels in rats. n = 10 in each group. **P < 0.01. **B** Detection of blood glucose level in rats after insulin injection. n = 10 in each group. **P < 0.01. **C** Representative echocardiographic images of the rat hearts. **D**-**H** Evaluation of the mean values of LVIDs, LVAWs, LVPWs, EF, and FS in rats. n = 10 in each group. **P < 0.01. **I** Determination of the heart weight/body weight (HW/BW) ratio in rats. n = 10 in each group. **P < 0.01. **J** Representative images of H & E staining in rat heart tissues from control and T2DM groups. **K** Representative images of Masson’s staining in rat left ventricular tissues. Scale bar = 50 μm. **L** Screening of oxidative stress-responsive miRNAs in rat left ventricle tissues. n = 5 for each group. *P < 0.05, and ** *P* < 0.01 vs. control group. **M** Relative expression level of miR-92a-2-5p in rat left ventricular tissues. n = 10 in each group. ** *P* < 0.01

Experimental T2DM rats were established by HFD and STZ treatment to confirm the miRNA expression profile in DCM hearts. After 8-week modeling, elevated blood glucose levels and impaired insulin sensitivity were detected in rats from T2DM group (Fig. 1A, B). Cardiac echocardiography showed that the cardiac structure and function of T2DM rats were markedly disordered (Fig. 1C). Compared with the control group, the mean values for left ventricular systolic anterior wall thickness (LVAWs), left ventricular systolic posterior wall thickness (LVPWs), and left ventricular systolic inner diameter (LVIDs) were significantly increased in the T2DM group (Fig. 1D–F), while the mean value of EF and FS were significantly decreased in the T2DM group (Fig. 1G, H). Moreover, significant myocardial hypertrophy and fibrosis were observed in the hearts of T2DM rat (Fig. 1I–K, and Additional file 1: Figure S1). These results suggest that DCM develops in T2DM rats. Ten oxidative stress-responsive miRNAs were selected as candidates based on the previous studies [29–36]. Through screening (Fig. 1L) and validation (Fig. 1M), qRT-PCR assay confirmed that the expression of miR-92a-2-5p was significantly decreased in the T2DM group, which was only 31.8% of that in the control group.

### Reduced expression of miR-92a-2-5p was detected in cardiomyocyte oxidative stress injury

**Fig. 2:**
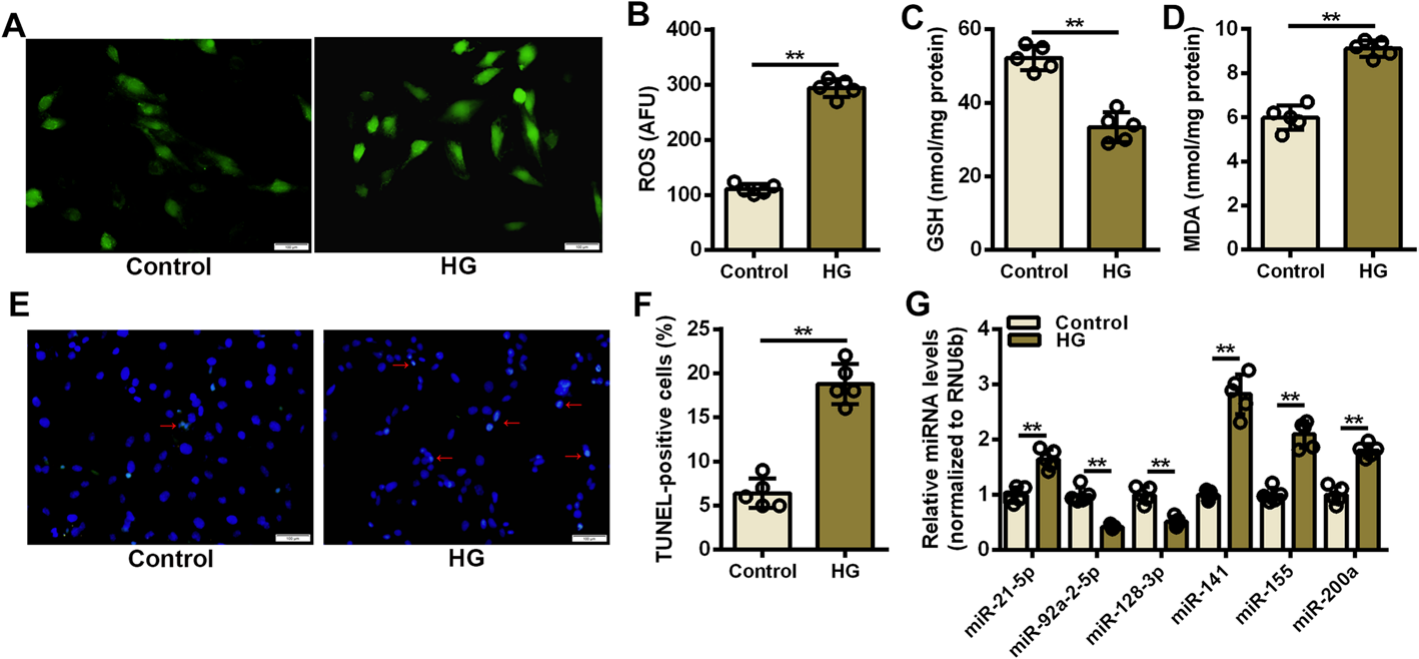
Reduced expression of miR-92a-2-5p was detected in cardiomyocyte oxidative stress injury. **A** Representative images of DCFH-DA staining of cardiomyocytes stimulated by high glucose. **B**-**D** Detection of ROS, GSH, and MDA levels in cardiomyocytes stimulated with high glucose. n = 5 in each group. **P < 0.01. **E** Representative images of TUNEL staining in cardiomyocytes stimulated by high glucose. The red arrows indicate the TUNEL-DAPI merge signals. **F** Detection of TUNEL-positive cardiomyocytes after high glucose stimulation. n = 5 in each group. **P < 0.01. **G** Relative expression levels of oxidative stress-responsive miRNAs in high glucose-stimulated cardiomyocytes. n = 5 in each group. ** *P* < 0.01 vs. control group

Myocardial oxidative stress injury is an important pathological manifestation of DCM. Therefore, the expression of these miRNAs was examined in cardiomyocyte oxidative stress injury. After high glucose stimulation for 48 h, intracellular ROS levels were significantly increased in cardiomyocytes (Fig. 2A, B). Decreased levels of the antioxidant glutathione (GSH) and increased accumulation of the oxidative stress marker malondialdehyde (MDA) were also detected in high glucose-stimulated cardiomyocytes (Fig. 2C, D). The TUNEL assay showed a threefold increase in TUNEL-positive cardiomyocytes in the high glucose group compared with the control group (Fig. 2E, F and Additional file 1: Figure S2). The results of qRT-PCR assay confirmed that the expression of miR-92a-2-5p was significantly decreased in cardiomyocytes stimulated by high glucose (Fig. 2G).

### Overexpression of miR-92a-2-5p attenuated oxidative stress injury in cardiomyocytes

**Fig. 3:**
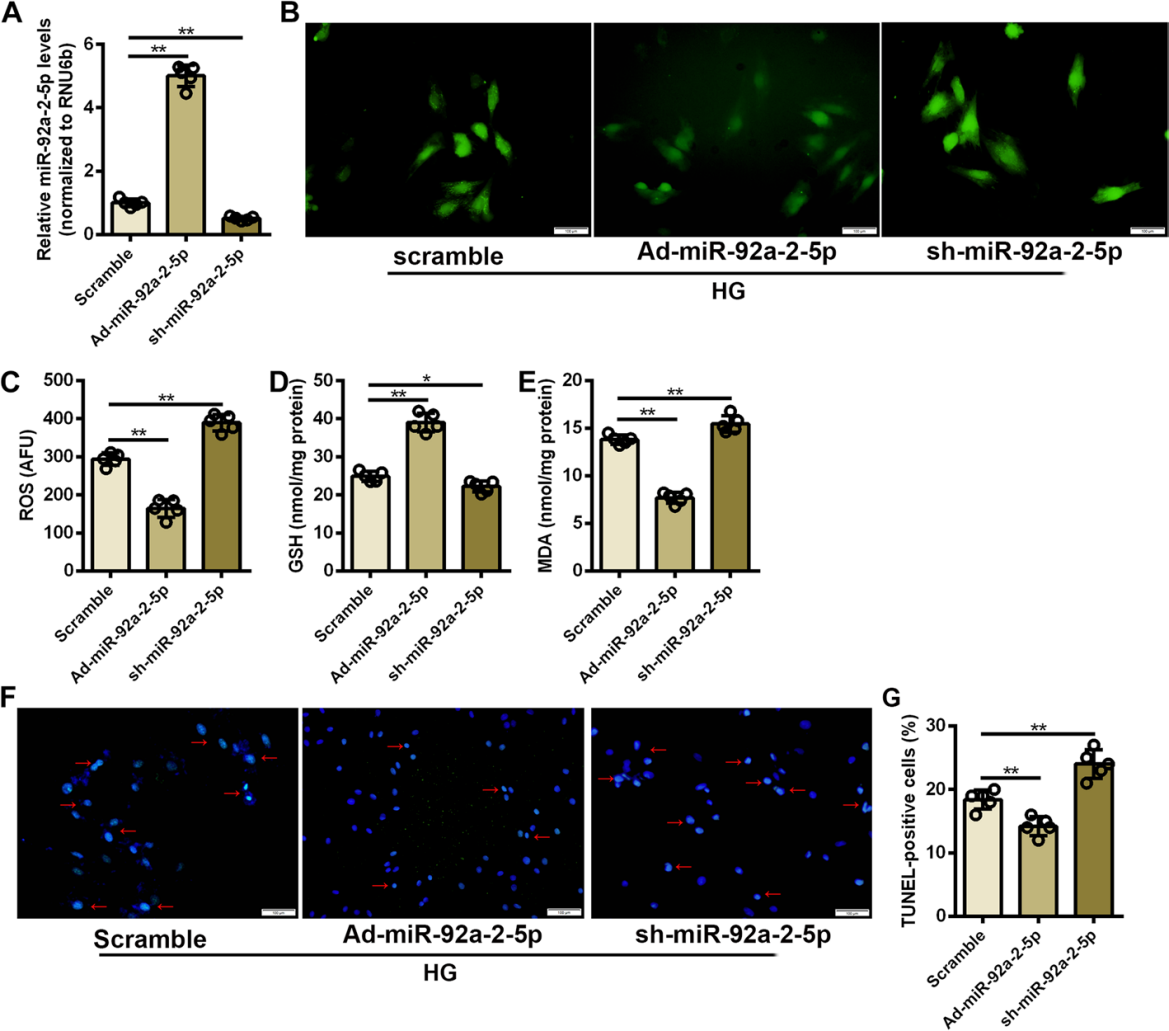
Overexpression of miR-92a-2-5p attenuated oxidative stress injury in cardiomyocytes. **A** Relative expression levels of miR-92a-2-5p in cardiomyocytes infected with adenovirus. n = 5 in each group. **P < 0.01. **B** Representative images of DCFH-DA staining in cardiomyocytes infected with adenovirus under high glucose stimulation. **C**-**E** Detection of ROS, GSH, and MDA levels in cardiomyocytes from the Ad-miR-92a-2-5p and sh-miR-92a-2-5p groups. n = 5 in each group. **P < 0.01. **F** Representative images of TUNEL staining in cardiomyocytes infected with adenovirus under high glucose stimulation. The red arrows indicate the TUNEL-DAPI merge signals. **G** Detection of TUNEL-positive cardiomyocytes in the Ad-miR-92a-2-5p and sh-miR-92a-2-5p groups. n = 5 in each group. **P < 0.01

Gain- and loss-of-function experiments were performed in cardiomyocytes under high glucose stimulation to investigate the role of miR-92a-2-5p in cardiomyocyte oxidative stress injury. The qRT-PCR results confirmed that the expression of miR-92a-2-5p was significantly increased in cardiomyocytes infected with Ad-miR-92a-2-5p (Fig. 3A). Overexpression of miR-92a-2-5p significantly reduced the accumulation of intracellular ROS in cardiomyocytes under high glucose stimulation (Fig. 3B, C). The level of GSH was significantly increased in cardiomyocytes after miR-92a-2-5p overexpression (Fig. 3D), while the level of MDA was significantly decreased (Fig. 3E). Moreover, oxidative stress-induced cardiomyocyte apoptosis was also significantly reduced by overexpression of miR-92a-2-5p, as demonstrated by the decreased number of TUNEL-positive cardiomyocytes in Ad-miR-92a-2-5p group (Fig. 3F, G, and Additional file 1: Figure S3). In contrast, infection with sh-miR-92a-2-5p significantly decreased the expression level of miR-92a-2-5p in cardiomyocytes, and exacerbated high glucose-induced oxidative stress injury and apoptosis in cardiomyocytes (Fig. 3A–G, and Additional file 1: Figure S3).

### MKNK2 was a novel target of miR-92a-2-5p in cardiomyocytes

**Fig. 4:**
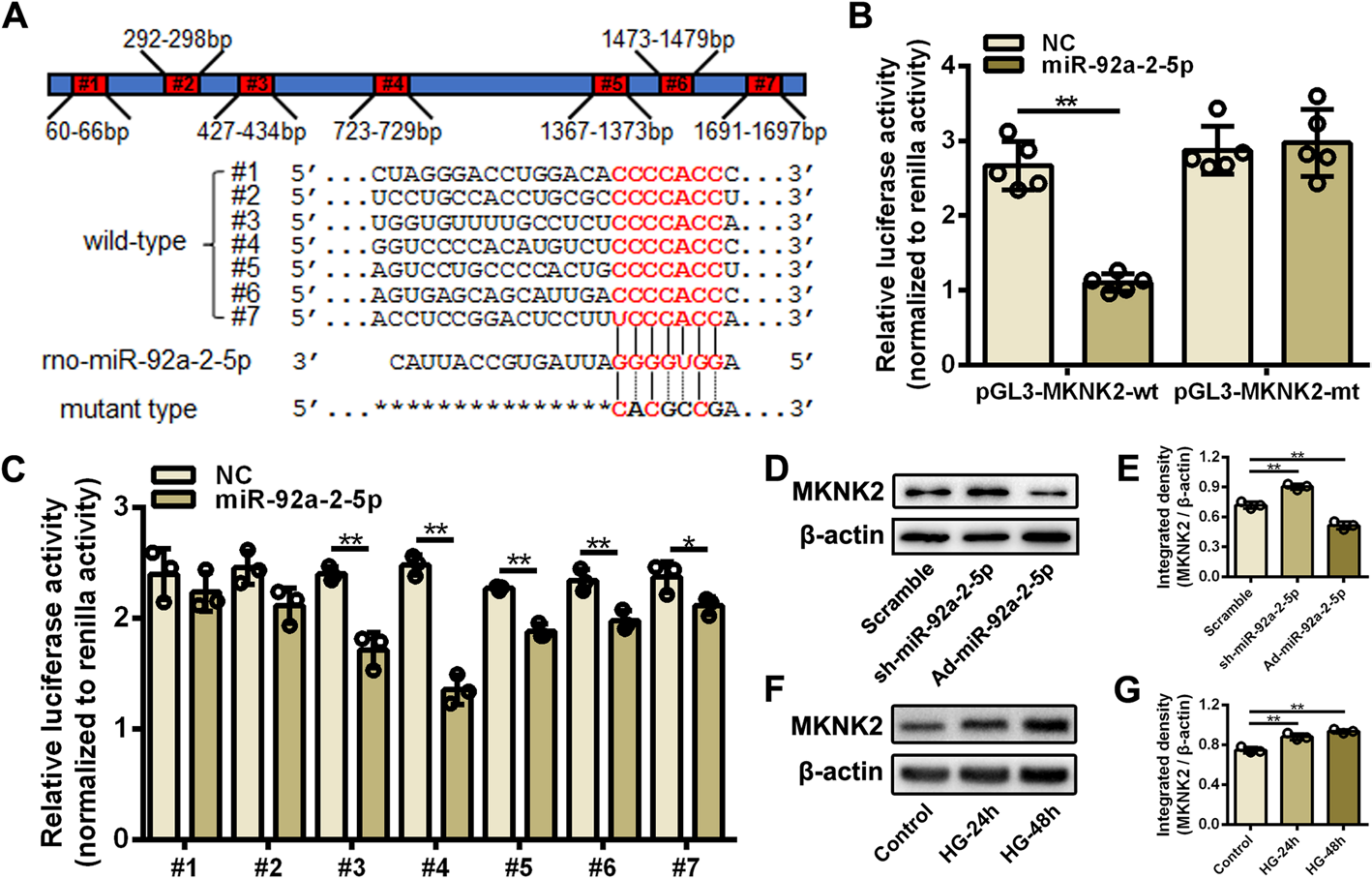
MKNK2 was a novel target of miR-92a-2-5p in cardiomyocytes. **A** Schematic representation of the full-length 3’UTR of rat MKNK2 mRNA. #1—#7, potential binding sites of miR-92a-2-5p. **B**, **C** Dual-luciferase reporter assay verified the binding of miR-92a-2-5p and wild- or mutant‐type 3’-UTR of MKNK2. n = 3 in each group. **P < 0.01. **D** Representative images of western blot for MKNK2 protein in cardiomyocytes infected with sh-miR-92a-2-5p or Ad-miR-92a-2-5p. E, Integrated density analysis of immune bands using ImageJ software. β-actin was used as a loading control. n = 3 in each group. **P < 0.01. **F** Representative images of western blot for MKNK2 protein in cardiomyocytes after 24-h or 48-h high glucose stimulation. **G** Integrated density analysis of immune bands using ImageJ software. β-actin was used as a loading control. n = 3 in each group. **P < 0.01

It is well-known that miRNAs exert their regulatory effects by inhibiting target expression in mammalian cells. Therefore, potential targets of miR-92a-2-5p were predicted using multiple bioinformatics algorithms. Based on these results, MAPK interacting serine/threonine kinase 2 (MKNK2) was selected as a putative target of miR-92a-2-5p. There were seven potential binding sites for miR-92a-2-5p in the 3’-UTR of rat MKNK2 mRNA (Fig. 4A). The results of dual-luciferase reporter assay showed that overexpression of miR-92a-2-5p significantly decreased the activity of the luciferase reporter containing wild-type 3’-UTR sequences of MKNK2, but had no obvious effect on the activity of the luciferase reporter containing mutant sequences (Fig. 4B). MiR-92a-2-5p had a stronger inhibitory effect on the 4th binding site (Fig. 4C). Western blot analysis confirmed that the protein expression of MKNK2 was significantly decreased in cardiomyocytes after miR-92a-2-5p overexpression, but significantly increased after miR-92a-2-5p inhibition (Fig. 4D, E). The protein expression of MKNK2 was also significantly increased in cardiomyocytes stimulated with high glucose (Fig. 4F, G). These results confirmed that MKNK2 was a target of miR-92a-2-5p in cardiomyocytes.

### miR-92a-2-5p attenuated cardiomyocyte oxidative stress injury by targeting MKNK2

**Fig. 5:**
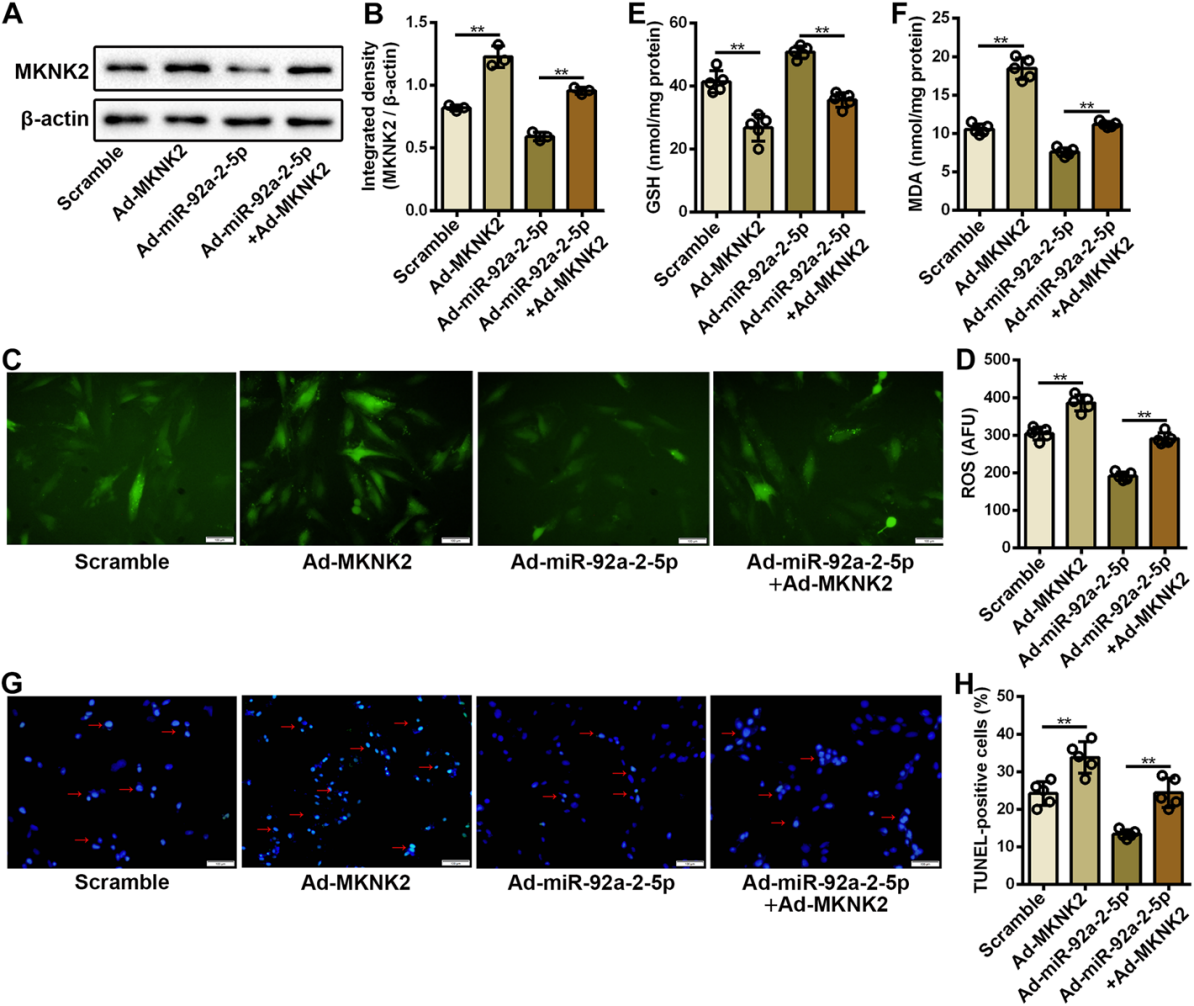
miR-92a-2-5p attenuated cardiomyocyte oxidative stress injury by targeting MKNK2. **A** Representative images of western blot for MKNK2 protein in cardiomyocytes infected with Ad-MKNK2 and/or Ad-miR-92a-2-5p under high glucose stimulation. **B** Integrated density analysis of immune bands using ImageJ software. β-actin was used as a loading control. n = 3 in each group. **P < 0.01. **C** Representative images of DCFH-DA staining in cardiomyocytes with adenovirus infection under high glucose stimulation. **D**-**F** Detection of ROS, GSH, and MDA levels in cardiomyocytes infected with adenovirus under high glucose stimulation. n = 5 in each group. **P < 0.01. **G** Representative images of TUNEL staining of cardiomyocytes infected with adenovirus under high glucose stimulation. The red arrows indicate the TUNEL-DAPI merge signals. **H** Detection of TUNEL-positive cardiomyocytes with adenovirus infection under high glucose stimulation. n = 5 in each group. **P < 0.01

To further confirm the effect of MKNK2 on oxidative stress injury in cardiomyocytes, gain-of-function experiments were performed in cardiomyocytes under high glucose stimulation. The western blot assay confirmed that protein expression of MKNK2 was significantly increased in cardiomyocytes after Ad-MKNK2 infection (Fig. 5A, B). High glucose-induced accumulation of intracellular ROS was significantly aggravated in cardiomyocytes after MKNK2 overexpression (Fig. 5C, D). Decreased GSH and increased MDA levels were also detected in cardiomyocytes infected with Ad-MKNK2 (Fig. 5E, F). The results from the TUNEL assay confirmed that high glucose-induced cardiomyocyte apoptosis was significantly increased after MKNK2 overexpression (Fig. 5G, H, and Additional file 1: Figure S4). Rescue experiments showed that the effect of miR-92a-2-5p on alleviating oxidative stress injury in cardiomyocytes could be abolished by MKNK2 overexpression, as demonstrated by changes in intracellular ROS, GSH, and MDA levels, and cardiomyocyte apoptosis (Fig. 5C–H, and Additional file 1: Figure S4).

### Administration of miR-92a-2-5p improved cardiac remodeling and function in diabetic rats

**Fig. 6:**
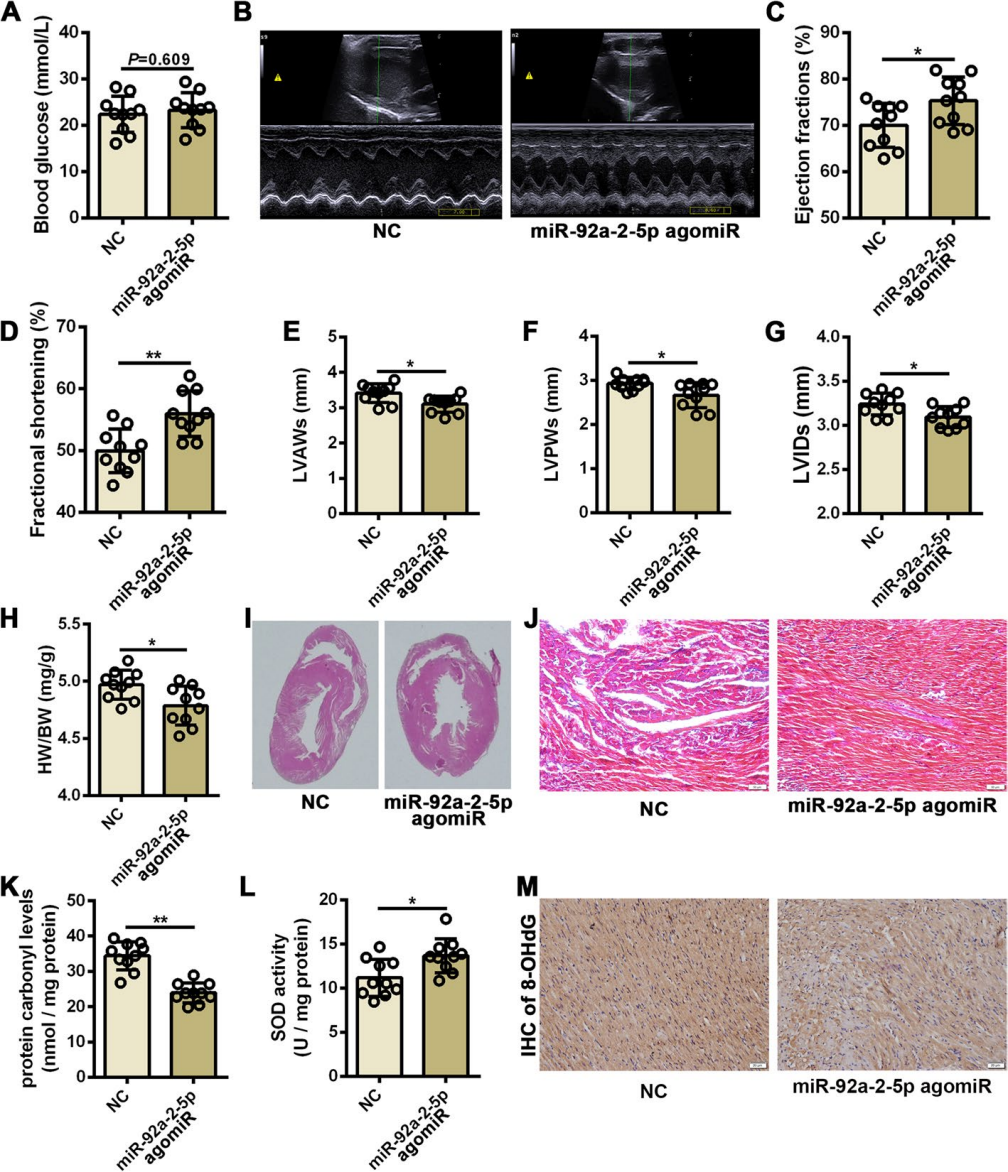
Administration of miR-92a-2-5p improved cardiac remodeling and function in diabetic rats. **A** Detection of random blood glucose level in diabetic rats injected with the miR-92a-2-5p agomiR. n = 10 in each group. **B** Representative echocardiographic images of the diabetic rat hearts from NC and miR-92a-2-5p agomiR groups. **C**-**G** Evaluation of the mean values of EF, FS, LVAWs, LVPWs, and LVIDs in diabetic rats. n = 10 in each group. *P < 0.05, and **P < 0.01. **H** Determination of heart weight/body weight (HW/BW) ratio in diabetic rats from the NC and miR-92a-2-5p agomiR groups. n = 10 in each group. **P < 0.01. **I** Representative images of H & E staining of rat hearts from NC and miR-92a-2-5p agomiR groups. **J** Representative images of Masson’s staining in rat left ventricle tissues from the NC and miR-92a-2-5p agomiR groups. Scale bar = 50 μm. **K**-**L** Detection of protein carbonyl levels and superoxide dismutase (SOD) activity in the heart tissues from the NC and miR-92a-2-5p agomiR groups. n = 10 in each group. *P < 0.05, and **P < 0.01. **M** Representative images of immunohistochemistry (IHC) for 8-OHdG in rat left ventricle tissues from the NC and miR-92a-2-5p agomiR groups. Scale bar = 20 μm

To determine whether miR-92a-2-5p could serve as a potential therapeutic target for DCM, an in vivo experiment was performed in diabetic rats through tail vein injection with miR-92a-2-5p agomiR. There was no significant difference in blood glucose levels between the NC and miR-92a-2-5p treated diabetic rats (Fig. 6A). However, compared to the NC group, the cardiac function of diabetic rats was significantly better in the miR-92a-2-5p group (Fig. 6B), as demonstrated by the increased EF and FS values (Fig. 6C, D). The disordered heart structure was also improved in the miR-92a-2-5p group, manifested as decreased values of LVAWs, LVPWs, and LVIDs (Fig. 6E–G). Pathological cardiac remodeling in diabetic rats was also significantly ameliorated after the administration of miR-92a-2-5p. Overexpression of miR-92a-2-5p antagonized the development of myocardial hypertrophy and fibrosis in diabetic rats (Fig. 6H–J, and Additional file 1: Figure S5). The protein carbonyl levels were significantly reduced in heart tissues from miR-92a-2-5p agomiR group (P < 0.01. Fig. 6K), while the activity of superoxide dismutase (SOD) was increased (P < 0.05. Fig. 6L). Immunohistochemical results confirmed that the signals of 8-OHdG was significantly reduced in heart tissues from miR-92a-2-5p agomiR group (Fig. 6M).

### miR-92a-2-5p modulated the p38-MAPK pathway by inhibiting MKNK2 expression

**Fig. 7:**
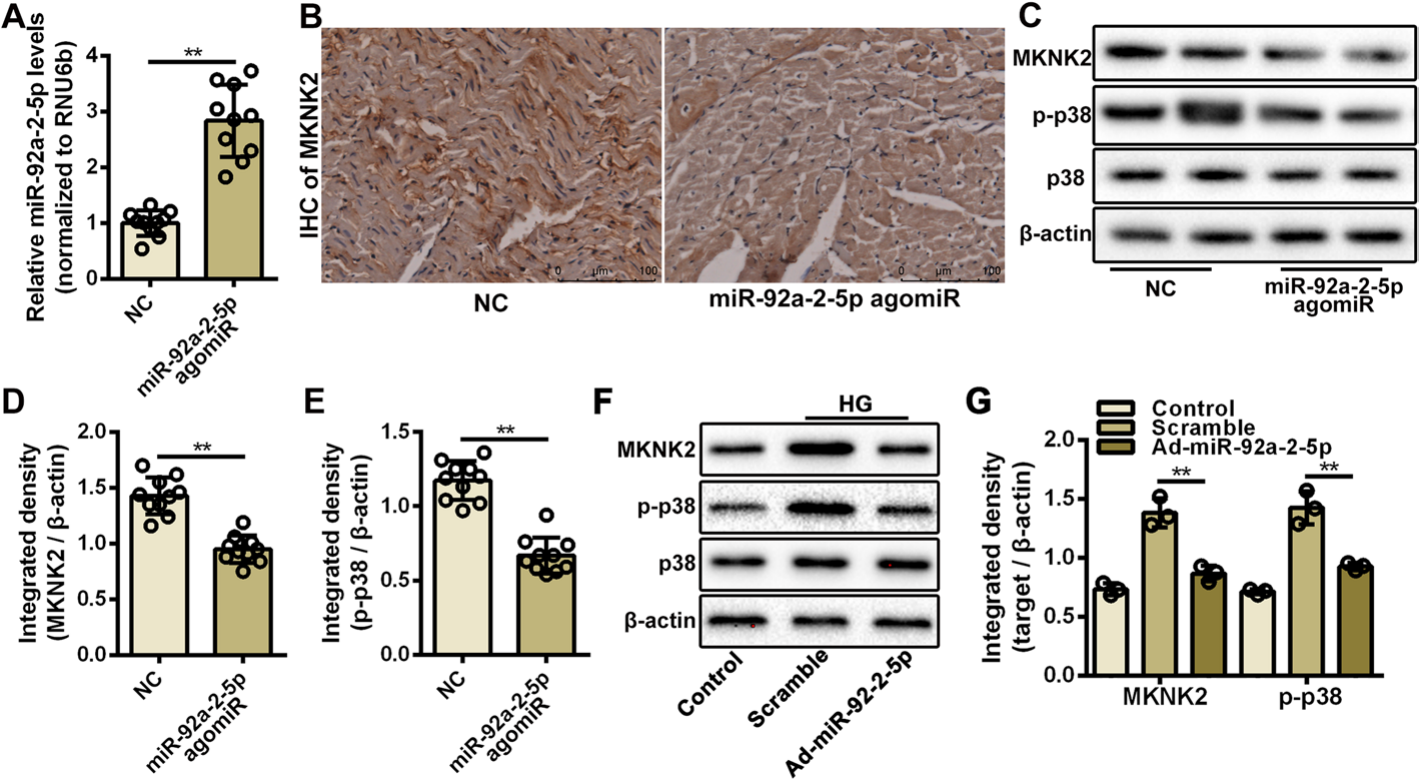
miR-92a-2-5p modulated the p38-MAPK pathway by inhibiting MKNK2 expression. **A** Relative expression level of miR-92a-2-5p in rat heart tissues from the NC and miR-92a-2-5p agomiR groups. ** *P* < 0.01. **B** Representative images of immunohistochemistry (IHC) for MKNK2 in rat left ventricle tissues from the NC and miR-92a-2-5p agomiR groups. Scale bar = 100 μm. **C** Representative images of western blot for MKNK2 and p38 proteins in NC and miR-92a-2-5p agomiR groups. **D**, **E** Integrated density analysis of immune bands using ImageJ software. β-actin was used as a loading control. n = 10 in each group. **P < 0.01. **F** Representative images of western blot for MKNK2 and p38 proteins in cardiomyocytes infected with Ad-miR-92a-2-5p under high glucose stimulation. **G** Integrated density analysis of immune bands using ImageJ software. β-Aactin was used as a loading control. n = 3 in each group. **P < 0.01

As an important serine/threonine kinase, MKNK2 has been identified to phosphorylate and activate the p38-MAPK pathway, thereby increasing cancer cell death [37, 38]. Therefore, the role of miR-92a-2-5p on activation of the p38-MAPK pathway was investigated during myocardial oxidative stress injury. Results from the qRT-PCR assay confirmed that miR-92a-2-5p levels in heart tissues were significantly elevated after injection with miR-92a-2-5p agomiR (Fig. 7A). Results from the immunohistochemistry assay showed that the expression of MKNK2 in heart tissues was significantly decreased in the miR-92a-2-5p group compared to that in the NC group (Fig. 7B), which was confirmed by the western blot assay (Fig. 7C, D). Decreased expression of phosphorylated p38 in heart tissues was also detected in the miR-92a-2-5p group (Fig. 7C, E). The role of miR-92a-2-5p on the activation of p38-MAPK pathway was also verified in cardiomyocytes. The western blot results showed that the expression of MKNK2 and phosphorylated p38 was significantly increased in cardiomyocytes treated with high glucose, but significantly decreased after overexpression of miR-92a-2-5p (Fig. 7F, G).

## Discussion

In the present study, we found that miR-92a-2-5p was downregulated in the heart tissues of diabetic rats and high glucose-induced cardiomyocytes. Administration of miR-92a-2-5p improved cardiac remodeling and function in diabetic rats. Furthermore, MKNK2 was identified as a novel target of miR-92a-2-5p in cardiomyocytes. Overexpression of miR-92a-2-5p attenuated high glucose-induced cardiomyocyte oxidative stress injury by inhibiting MKNK2-mediated activation of the p38-MAPK pathway.

During the initiation of this study, differential expression of several oxidative stress-responsive miRNAs was detected in vivo and in vitro. Some miRNAs were involved in the pathogenesis of DCM, such as miR-141 and miR-155 [39, 40]. The expression of miR-92a-2-5p was decreased in cardiomyocytes from DCM and treated with high glucose. However, the level of miR-92a-2-5p was significantly increased in the plasma of T2DM rats and in the cardiomyocyte medium with high glucose. MiR-92a-2-5p could enhance mitochondrial translation to reduce ROS production in cardiomyocytes [41], but the role of miR-92a-2-5p in the cardiomyocyte cytoplasm has not been investigated. Therefore, miR-92a-2-5p was selected as the target miRNA for this study.

Among these selected miRNAs, miR-128-3p has been reported to be involved in the regulation of various heart diseases. The expression of miR-128-3p in the myocardium was found to be increased during cardiac hypertrophy and heart failure [42, 43], while decreased during ischemia–reperfusion cardiac injury [44]. In the present study, the decreased miR-128-3p level was also detected in the heart tissues of T2DM rats, but its role in DCM has not been explored. It was demonstrated that reduction of miR-128-3p level would preserve insulin-stimulated glucose uptake in cardiomyocytes, and mitigate myocardial insulin resistance [43]. Therefore, the decreased expression of miR-128-3p in T2DM rat heart might be a protective mechanism against stress high glucose.

Typically, miRNAs serve as negative regulators of protein translation in the cytoplasm, but some miRNAs regulate gene expression positively in the mitochondria [45, 46]. During myogenesis, miR-1 was induced, entered into the mitochondria, and stimulated the translation of specific mitochondrial genome-encoded transcripts [45]. MiR-92a-2-5p and let-7b-5p could facilitate the translation of mitochondrial genes. Overexpression of miR-92a-2-5p or let-7b-5p could positively modulate cytochrome-b (mt-Cytb) expression, and reduce ROS production in cardiomyocytes [41]. The present study confirmed the translation inhibitory effect of miR-92a-2-5p in the cytoplasm. Overexpression of miR-92a-2-5p negatively regulated MKNK2 expression, and reduced ROS accumulation in cardiomyocytes. These results suggest that miR-92a-2-5p-mediated translational stimulation in the mitochondria and repression in the cytoplasm may be a highly coordinated program for cardiomyocyte oxidative stress.

MKNK2, one of the two serine/threonine kinases, is a substrate in the MAPK pathway. Elevated expression of MKNK2 has been identified in multiple types of tumors, including melanoma, prostate cancer, and soft tissue sarcoma [47–49]. Some selective inhibitors of MKNK2 have entered clinical trials, and display significant activity against several cancers [50]. Recent studies have found that disabling MKNK2 could protect against diet-induced obesity, which might result from greater ATP consumption, mitochondrial oxidative metabolism, and other energy utilization processes [51]. Moreover, MKNK2-deficient male mice exhibit protection against HFD-induced obesity and insulin resistance [52]. In the present study, inhibition of MKNK2 by miR-92a-2-5p improved cardiac remodeling and function in T2DM rats. These results suggest that the development of MKNK2-targeted therapy might be a useful treatment for reducing myocardial damage in DCM.

The present study identified seven potential binding sites for miR-92a-2-5p in the 3’UTR of rat MKNK2 mRNA. The results of dual-luciferase reporter assay confirmed that miR-92a-2-5p had clear inhibitory effects on the five binding sites. In contrast to rat MKNK2, human MKNK2 is alternatively spliced to produce two isoforms-MKNK2a and MKNK2b [53]. Compared to the MKNK2b isoform, the MKNK2a isoform has a longer 3’ coding region and 3’ UTR, and has a MAP kinase-binding region at the C-terminus. It has been identified that the MKNK2a isoform could activate the p38-MAPK pathway to induce apoptosis, while the MKNK2b isoform does not have this effect [38]. Bioinformatics screening showed that there were two potential binding sites for miR-92a-2-5p in the 3’UTR of the human MKNK2a isoform (Additional file 1: Figure S6), while no binding site was found in the MKNK2b isoform. These data suggest that miR-92a-2-5p may regulate the expression of MKNK2a in humans, and may be a key regulator of the balance between MKNK2a and MKNK2b expression. It would be interesting and important to investigate the role of miR-92a-2-5p in human MKNK2 expression in the future.

Myocardial fibrosis is the main characteristic of cardiac structural remodeling, and is an inevitable process during the development of various cardiovascular diseases. Myocardial fibrosis is closely related to arrhythmia, cardiac dysfunction and even sudden cardiac death [54]. Excessive oxidative stress could cause accelerated fibrosis in the diabetic heart, thus leading to severe cardiac dysfunction [55]. Therefore, decreasing ROS production to reduce oxidative stress has emerged as a potential therapeutic strategy for DCM. In the present study, the improvement of myocardial fibrosis was also observed in DCM rats with miR-92a-2-5p treatment, suggesting its potential role as the anti-oxidant therapeutic strategy for protecting against fibrosis in DCM.

## Conclusions

The present study demonstrated the role of miR-92a-2-5p in myocardial damage during DCM development. Overexpression of miR-92a-2-5p inhibited the expression of MKNK2 in cardiomyocytes, leading to decreased phosphorylation of p38-MAPK signaling, which in turn, attenuated high glucose-induced cardiomyocyte oxidative stress injury. Additionally, in vivo experiments showed that the administration of miR-92a-2-5p could improve cardiac remodeling and function in diabetic rats. These results suggest that miR-92a-2-5p may be a potential therapeutic target for the prevention of myocardial damage in DCM.

## Supplementary Information

**Additional file 1.** Supplementary Tables and Figures.

## Data Availability

The datasets used and analyzed during the current study are available from the corresponding author on reasonable request.

## Abbreviations

DCM: Diabetic cardiomyopathy
DM: Diabetes mellitus
ROS: Reactive oxygen species
MAPK: Mitogen-activated protein kinase
miRNA: MicroRNA
SD: Sprague–Dawley
HFD: High-fat diet
STZ: Streptozotocin
H&E: Hematoxylin and eosin
DCFH-DA: 2′,7′-Dichlorofluorescein diacetate
DAB: 3,3′-Diaminobenzidine
BrdU: Bromodeoxyuridine
DCFH-DA: 2′,7′-Dichlorofluorescein diacetate
PBS: Phosphate-buffered saline
DAPI: 4′,6-Diamidino-2-phenylindole
pfu: Plaque-forming units
MOI: Multiplicity of infection
qRT-PCR: Quantitative real-time PCR
BCA: Bicinchoninic acid
PVDF: Polyvinylidene fluoride
LVAWs: Left ventricular systolic anterior wall thickness
LVPWs: Left ventricular systolic posterior wall thickness
LVIDs: Left ventricular systolic inner diameter
GSH: Glutathione
MDA: Malondialdehyde
MKNK2: MAPK interacting serine/threonine kinase 2
