# Supplementary material for: Therapeutic overexpression of miR-92a-2-5p ameliorated cardiomyocyte oxidative stress injury in the development of diabetic cardiomyopathy
Source: Cell Mol Biol Lett. 2022 Oct 8;27:85. doi: 10.1186/s11658-022-00379-9 (PMC9548149; doi:10.1186/s11658-022-00379-9)
Supplement: Supplementary file 1 — Additional file 1. Supplementary Tables and Figures. [file 11658_2022_379_MOESM1_ESM.docx]

**Supplemental data**

Tab. S1 The sequences of primers used in the qRT-PCR assay

| Gene name | Sequences of primer (5’-3’) |
| --- | --- |
| miR-92a-2-5p | Stem-loop: CTCAACTGGTGTCGTGGAGTCGGCAATTCAGTTGAGTCCACCCC  Forward: ACACTCCAGCTGGGCATTACCGTGATTAGG |
| miR-9-5p | Stem-loop: CTCAACTGGTGTCGTGGAGTCGGCAATTCAGTTGAGTCATACAG  Forward: ACACTCCAGCTGGGTCTTTGGTTATCTAGCT |
| miR-21-5p | Stem-loop: CTCAACTGGTGTCGTGGAGTCGGCAATTCAGTTGAGTCAACATC  Forward: ACACTCCAGCTGGGTAGCTTATCAGACTGA |
| miR-128-3p | Stem-loop: CTCAACTGGTGTCGTGGAGTCGGCAATTCAGTTGAGAAAGAGAC  Forward: ACACTCCAGCTGGGTCACAGTGAACCGGT |
| miR-141 | Stem-loop: CTCAACTGGTGTCGTGGAGTCGGCAATTCAGTTGAGCCATCTTT  Forward: ACACTCCAGCTGGGTAACACTGTCTGGTAA |
| miR-144 | Stem-loop: CTCAACTGGTGTCGTGGAGTCGGCAATTCAGTTGAGAGTACATC  Forward: ACACTCCAGCTGGGTACAGTATAGATGA |
| miR-146a | Stem-loop: CTCAACTGGTGTCGTGGAGTCGGCAATTCAGTTGAGAACCCATG  Forward: ACACTCCAGCTGGGTGAGAACTGAATTCCA |
| miR-153 | Stem-loop: CTCAACTGGTGTCGTGGAGTCGGCAATTCAGTTGAGGATCACTT  Forward: ACACTCCAGCTGGGTTGCATAGTCACAAAA |
| miR-155 | Stem-loop: CTCAACTGGTGTCGTGGAGTCGGCAATTCAGTTGAGACCCCTAT  Forward: ACACTCCAGCTGGGTTAATGCTAATCGTGAT |
| miR-200a | Stem-loop: CTCAACTGGTGTCGTGGAGTCGGCAATTCAGTTGAGACATCGTT  Forward: ACACTCCAGCTGGGTAACACTGTCTGGTAA |
| universal reverse primer | CTCAACTGGTGTCGTGGAGT |

Tab. S2 The application information of antibodies used in the experiments

| Name | Description | Company information | Application information |
| --- | --- | --- | --- |
| Anti-MKNK2 | Rabbit polyclonal | Proteintech, Wuhan, China | 1:500 dilution in WB;  1:200 dilution in IHC |
| Anti-8-OHdG | Mouse monoclonal | Santa Cruz, CA, USA | 1:100 dilution in IHC |
| Anti-p38 | Rabbit monoclonal | Abcam, Cambridge, UK | 1:1000 dilution in WB |
| Anti-p-p38 | Rabbit polyclonal | Abcam, Cambridge, UK | 1:1000 dilution in WB |
| Anti-β-actin | Mouse monoclonal | Abcam, Cambridge, UK | 1:2000 dilution in WB |


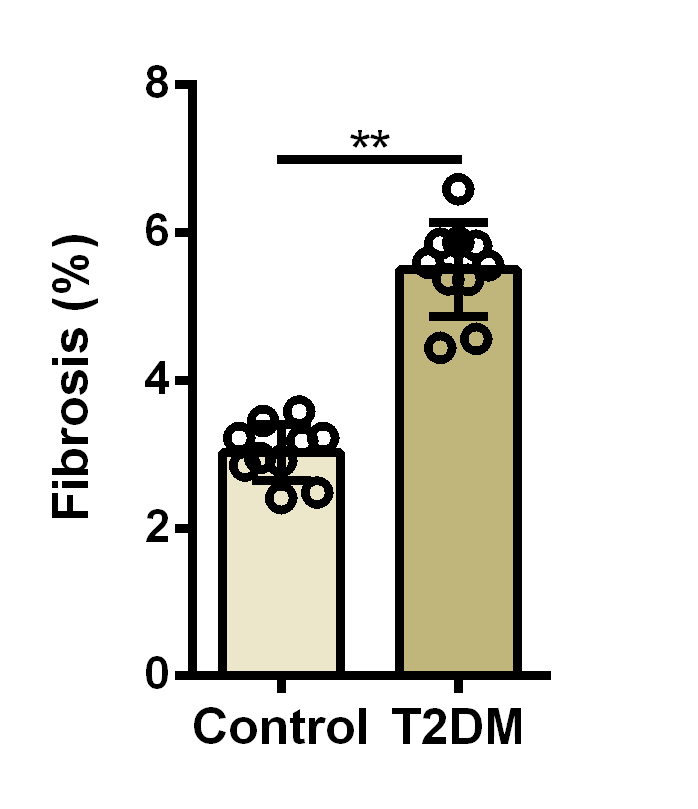


Figure S1 Quantification of fibrotic areas in DCM hearts. Fibrotic area in the section of heart tissue was quantified by ImageJ software. n=10 in each group.


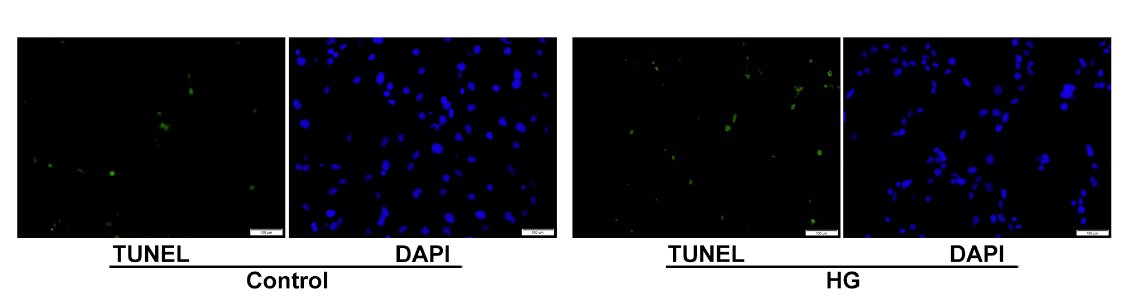


Figure S2 TUNEL- and DAPI-staining of cardiomyocytes under high glucose stimulation. TUNEL signals are green, DAPI signals are blue. HG, high glucose (33 mmol/L).


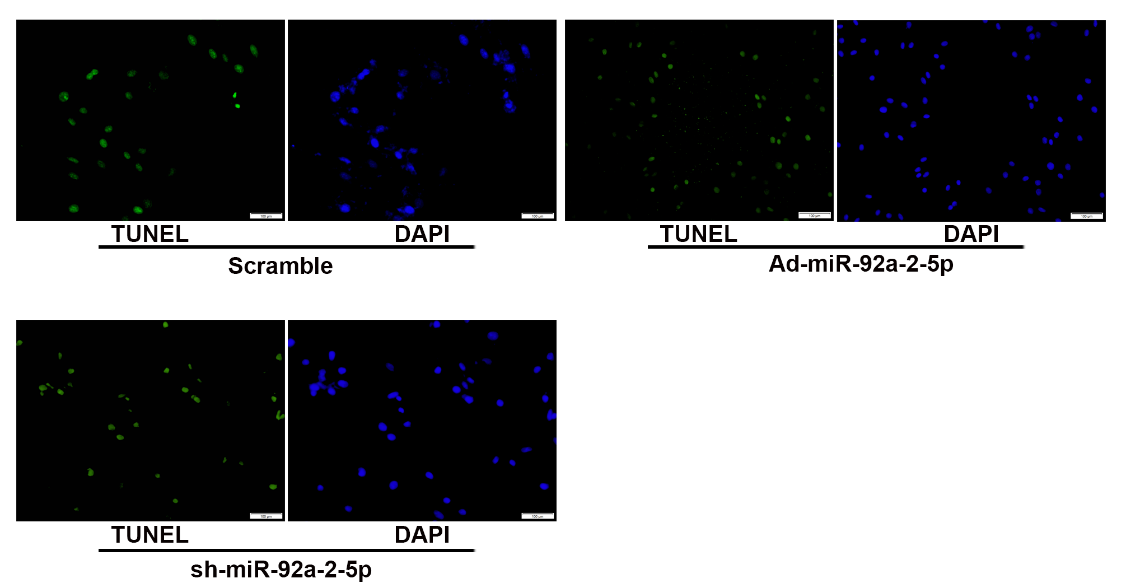


Figure S3 TUNEL- and DAPI-staining of cardiomyocytes with miR-92a-2-5p overexpression or inhibition under high glucose stimulation. TUNEL signals are green, DAPI signals are blue.

**
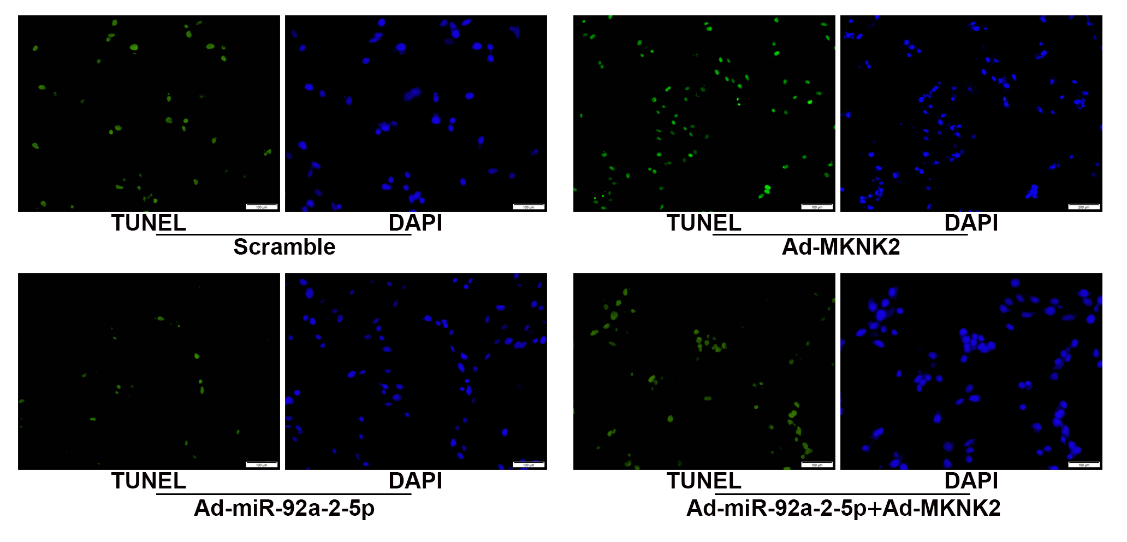
**

Figure S4 TUNEL- and DAPI-staining of cardiomyocytes with miR-92a-2-5p/MKNK2 expression under high glucose stimulation. TUNEL signals are green, DAPI signals are blue.


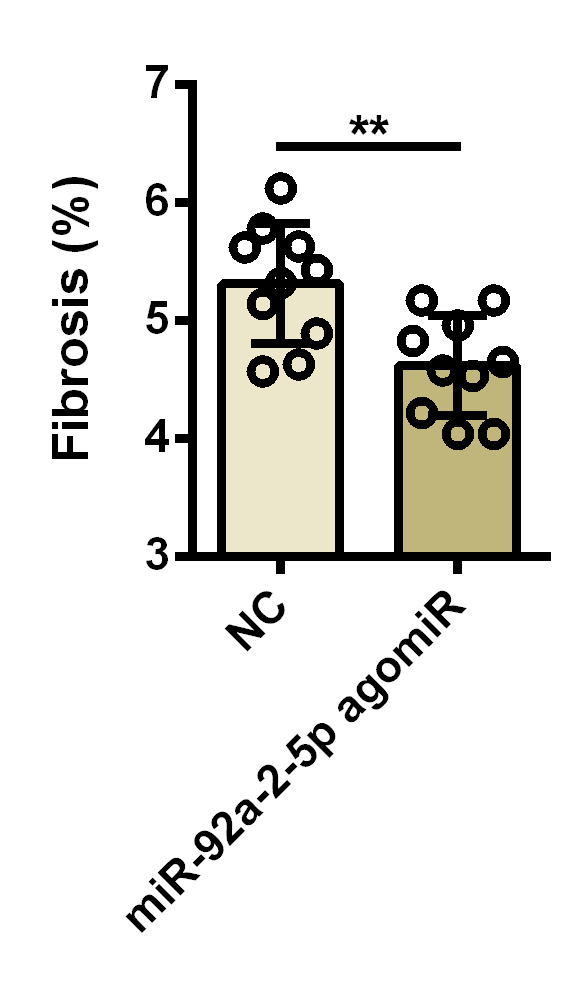


Figure S5 Quantification of fibrotic areas in DCM hearts with miR-92a-2-5p agomiR treatment. Fibrotic area in the section of heart tissue was quantified by ImageJ software. n=10 in each group.


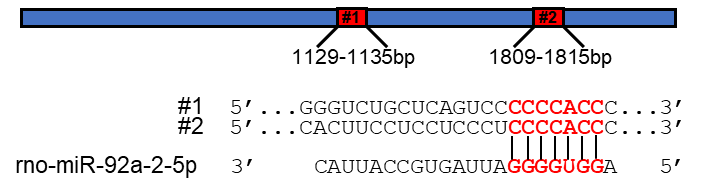


Fig. S6 The schematic of full-length 3’UTR information of human MKNK2a isoform. #1-#2, the potential binding sites of miR-92a-2-5p.
